# Characterization of Environmental and Cultivable Antibiotic-Resistant Microbial Communities Associated with Wastewater Treatment

**DOI:** 10.3390/antibiotics10040352

**Published:** 2021-03-26

**Authors:** Alicia Sorgen, James Johnson, Kevin Lambirth, Sandra M. Clinton, Molly Redmond, Anthony Fodor, Cynthia Gibas

**Affiliations:** 1 Department of Biological Sciences, University of North Carolina at Charlotte, Charlotte, NC 28223, USA; asorgen@uncc.edu (A.S.); mollyredmond@gmail.com (M.R.); 2 Department of Bioinformatics and Genomics, University of North Carolina at Charlotte, Charlotte, NC 28223, USA; jjohn443@uncc.edu (J.J.); kclambirth@uncc.edu (K.L.); afodor@uncc.edu (A.F.); 3 Department of Geography & Earth Sciences, University of North Carolina at Charlotte, Charlotte, NC 28223, USA; sclinto1@uncc.edu

**Keywords:** culturability, antibiotic resistance, wastewater treatment

## Abstract

Bacterial resistance to antibiotics is a growing global concern, threatening human and environmental health, particularly among urban populations. Wastewater treatment plants (WWTPs) are thought to be “hotspots” for antibiotic resistance dissemination. The conditions of WWTPs, in conjunction with the persistence of commonly used antibiotics, may favor the selection and transfer of resistance genes among bacterial populations. WWTPs provide an important ecological niche to examine the spread of antibiotic resistance. We used heterotrophic plate count methods to identify phenotypically resistant cultivable portions of these bacterial communities and characterized the composition of the culturable subset of these populations. Resistant taxa were more abundant in raw sewage and wastewater before the biological aeration treatment stage. While some antibiotic-resistant bacteria (ARB) were detectable downstream of treated wastewater release, these organisms are not enriched relative to effluent-free upstream water, indicating efficient removal during treatment. Combined culture-dependent and -independent analyses revealed a stark difference in community composition between culturable fractions and the environmental source material, irrespective of culturing conditions. Higher proportions of the environmental populations were recovered than predicted by the widely accepted 1% culturability paradigm. These results represent baseline abundance and compositional data for ARB communities for reference in future studies addressing the dissemination of antibiotic resistance associated with urban wastewater treatment ecosystems.

## 1. Introduction

Antibiotics are antimicrobial substances that can either kill or inhibit the growth and replication of a bacterium [1]. Antibiotics have revolutionized the field of medicine; yet their increased use has exerted a selective pressure on susceptible bacteria, favoring the survival of antibiotic-resistant bacteria (ARB) and the proliferation of associated antibiotic resistance genes (ARGs) [2]. Some bacterial species are inherently resistant to certain antibiotics as a result of structural or functional characteristics [3,4]. Those not inherently resistant can acquire resistance through mutations in chromosomal genes and via horizontal gene transfer (HGT) furthering the development of resistance among previously susceptible organisms [5].

Wastewater treatment plants (WWTP) are major sewage repositories that may receive sewage from both residential and medical treatment facilities. The continuous inflow of pre-existing ARB and antibiotic residues are important sources of resistance material [6,7]. The wastewater treatment process generally involves three stages of treatment where (1) large solids are removed through physical processes before (2) entering a sedimentation tank in which remaining suspended solids sink to form activated sludge and where biological and chemical processes are employed to remove organic matter [6]. Any additional organic or chemical components are removed using (3) additional treatment processes such as chemical and biological-chemical contact filters [8,9]. Prior to environmental release, wastewater undergoes a disinfection stage to eliminate harmful bacteria in the effluent [10]. WWTPs are considered “hotspots” for resistance dissemination due to the sub-inhibitory concentrations of antibiotic compounds and the favorable conditions that promote HGT [11,12,13]. Insight into ARB community trends throughout the treatment process can help us understand the effect of released treated wastewater on natural microbial communities [11,12,13].

Areas with limited water treatment infrastructure have been shown to pass human microbial resistomes into the environment via mobile genetic elements (MGEs), such as plasmids, transposons, and integrons [14,15]. Even in locations with innovative wastewater treatment systems, the degree of contaminant removal depends on the particular technology and operating parameters used, whether it be UV, chlorine, or microfiltration disinfection methods [16].

**Figure 1:**
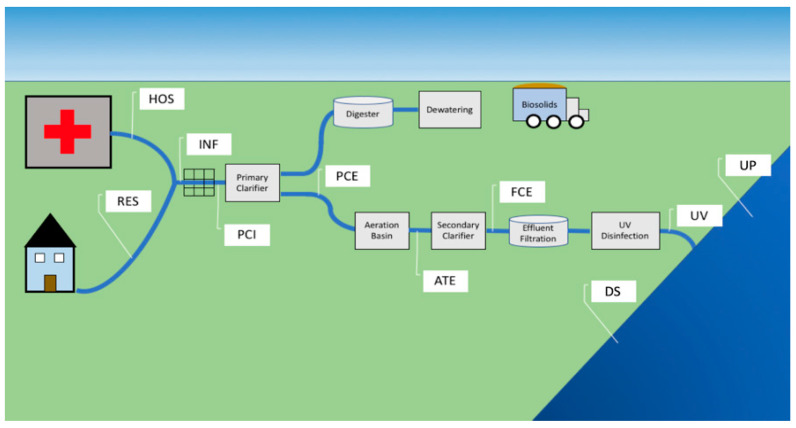
Schematic of the wastewater treatment process and sampling sites utilized for this study. Raw sewage from residential (RES) and hospital (HOS) sources are routed to a main sewer line. The combined raw influent (INF) is then passed through a physical screen to filter out large solids. The screen-filtered wastewater (PCI) undergoes primary clarification. The primary clarifier effluent (PCE) is routed to an aeration basin for biological nutrient removal. The aeration tank effluent (ATE) then undergoes a secondary clarification process. The final clarification effluent (FCE) undergoes a final filtration step before UV treatment (UV) for microbial disinfection prior to stream release. The large solids removed during primary clarification are routed to a digester and dewatered for the production of biosolids. (Downstream, DS; Upstream, UP).

In this study, we sampled sites along WWTP-associated urban streams. We sampled stream locations upstream (UP) and downstream (DS) of the treated wastewater release point to understand the overall impact of treated water release on the stream microbial community (Figure 1). Inside the WWTP, we sampled untreated sewage influent and the effluent of each treatment stage to identify which taxa are introduced to the WWTP via influent and whether they are subsequently completely removed. Samples pulled at each site within the stream environment and within the WWTP (as described in Lambirth et al., 2018) were characterized using 16S rRNA amplicon and metagenomic sequencing.

In parallel, each sample was cultured, and heterotrophic plate count methods were used to isolate antibiotic-resistant subpopulations from treated wastewater and streamwater samples. We then used 16S rRNA amplicon sequencing to characterize these resistant subpopulations and compare them to the source environment microbial communities. This experiment provides a window into potential antibiotic resistance that cannot be achieved by simply screening whole-microbiome sequencing data for resistant organisms or resistance signatures.

Heterotrophic plate count methods are often employed to culture and represent subpopulations of environmental samples, and as all commensals and opportunistic pathogens are heterotrophic, plate count methods are often performed to assess water quality [17]. Such an approach allows us to investigate phenotypic variations in subpopulations, including antibiotic resistance patterns. However, as described by “the great plate count anomaly”, not all of the heterotrophic microbial population can be easily cultivated from environmental samples [18]. Microbial communities are a consortium of many organisms each requiring specific conditions for survival and replication, which cannot be replicated under laboratory conditions as yet [19,20]. Aside from the physical niche required for an organism’s growth, it has been shown that some bacteria cannot grow unless their syntrophic counterparts are present for the exchange of essential growth factors [21,22,23].

The use of culture-independent techniques, such as those based on 16S rRNA gene sequencing, allows us to identify a large portion of the microbial population that cannot be observed by plate count methods [24], yet is limited in that it provides no information on bacterial resistance profiles. The use of such an approach alone offers no insight into the physiological and ecological roles played within the community [25]. Even metagenomic approaches only provide an understanding of the genetic potential of the community and as yet, many microbial genes remain uncharacterized [26].

The potential human impact on environmental surface water microbiomes in Charlotte, NC is significant as this is a city of approximately 870,000 people within a larger metropolitan area of 2.5 million [27]. We sampled bacterial communities from two WWTPs and their receiving surface water located in Charlotte. By combining traditional plate count and 16S rRNA gene sequencing methods to analyze these samples, we can (1) identify changes in resistant microbial communities throughout successive stages of the treatment process and (2) compare resistant communities identified via plate counts to that of the total environmental populations observed.

We cultured samples at both room and human body temperature in order to isolate resistant organisms that are culturable under normal conditions and organisms that can potentially withstand survival in the human body. By sequencing the cultured microbial communities using the same methodology used for culture-independent assessments of these same communities, we attempt to identify the various fractions of the environmental communities that are cultivable ARB, while also observing resistance patterns within the treatment process. By culturing and sequencing the microbial communities associated with wastewater treatment under various nutrient and temperature conditions with amendments of commonly prescribed antibiotics, we can culture representative communities of ARB.

## 2. Results

### 2.1. 16S rRNA Sequence Analysis Revealed a Pattern of Cultivable Taxa

We first examined and compared the overall compositional characteristics of the environmental and cultured microbial communities. The Illumina HiSeq 16S rRNA sequencing runs of the cultivated heterotrophic plate count and culture-independent environmental DNA samples yielded 179,945,081 and 24,601,764 sequence reads, respectively. The 227 cultivated plate count samples were represented by 959 unique operational taxonomic units (OTU) from the paired-end reads generated, while the 76 environmental samples were represented by 830 OTUs, of which 649 classified OTUs were recovered from both the environmental source material and cultured communities. The number of reads in each cultured sample ranged from 70,151 to 2,184,668 with an average of 792,710 ± 331,623 reads/sample with environmental samples ranging from 11,928 to 762,638 with a mean of 323,707 ± 175,629 reads/sample. The Shannon diversity index of the cultured samples had a mean value of 2.06 ± 0.87 while the environmental samples had a significantly higher mean value of 3.08 ± 0.75 (*p* < 0.001).

As a whole, the identified culture sequences were assigned to 87 unique genera, belonging to 60 families from nine phyla of the bacterial domain. At the phylum level, Proteobacteria dominated the community (89.17%), followed by lower proportions of Bacteroidetes (4.19%), Firmicutes (4.07%), and Actinobacteria (2.57%); representative OTUs were present in all sample locations. At the family level, the identified OTUs were predominantly classified as *Enterobacteriaceae* (32.16%) and *Pseudomonadaceae* (17.93%), which were found throughout all samples.

The environmental samples were assigned to 10 total phyla comprising 72 unique genera from 57 families. These samples were dominated by OTUs identified as Proteobacteria (62.39%) with lesser concentrations of Bacteroidetes (16.70%), Actinobacteria (14.04%), and Firmicutes (5.28%). *Comamonadaceae* (18.97%) was the most abundant Proteobacterial family followed by *Thiotrichaceae* (7.54%) and *Neisseriaceae* (6.63%), with the second most abundant family, *Sporichthyaceae* (7.90%), from the phylum Actinobacteria.

### 2.2. Similar Bacterial Communities Were Observed in Mallard and Sugar Creek WWTPs

In order to identify any differences between the two WWTP-associated stream systems, and to determine whether they could be combined for the purpose of subsequent analysis, we next compared both the heterotrophic plate count results and the 16S amplicon sequencing for the two systems. No differences in the abundance of bacterial colonies (*p* = 0.74; Supplementary Table S1) or the community structure identified via taxonomic diversity analyses (Supplementary S1; Table S2) were observed between the communities originating from samples taken inside the Mallard and Sugar Creek treatment plants. Linear regression analysis of the CFU/mL normalized total bacterial communities found within Mallard and Sugar Creek sites indicated no significant variation in the taxonomic abundance in the bacterial cultures from the two sampling sites (Supplementary Table S3).

### 2.3. The Treatment Process Significantly Reduces ARB Colony Counts

**Figure 2:**
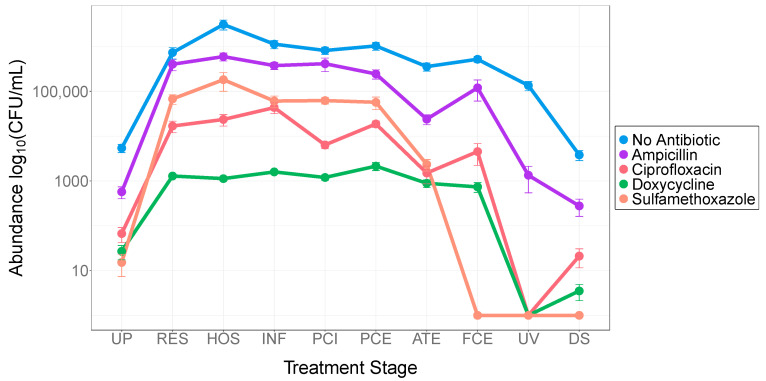
Abundance of microbial colonies grown on the four antibiotics and a control at each stage of the treatment process. Total heterotrophic and resistant microbial concentrations saw significant reductions from the initial raw sewage to the final UV-treated effluent. Error bars indicate the standard error of the mean for each antibiotic treatment at each site. UP, upstream; RES, residential sewage; HOS, hospital sewage; INF, sewage influent; PCI, primary clarification influent; PCE, primary clarification effluent; ATE, aeration tank effluent; FCE, final clarification effluent; UV, UV treated effluent; DS, downstream.

Analysis of antibiotic-resistant colony counts through each stage of the treatment process revealed a significant decrease in survival upon treatment (Figure 2). Raw sewage and the wastewater collected from preliminary treatment stages (RES, HOS, INF, PCI, and PCE) yielded the highest number of colonies per plate of total heterotrophic and antibiotic-resistant colonies (Supplementary Table S4). Antibiotic-resistant colony yields decreased significantly during the biological activated sludge treatment stage (ATE) when grown in the presence of antibiotics: ampicillin by 90.22% (*p* < 0.001), ciprofloxacin by 91.96% (*p* < 0.001), doxycycline by 58.49% (*p* = 0.001), and sulfamethoxazole by 95.88% (*p* < 0.001). Total heterotrophic growth did not appear to be affected during this stage of treatment (Supplementary Table S1).

**Figure 3:**
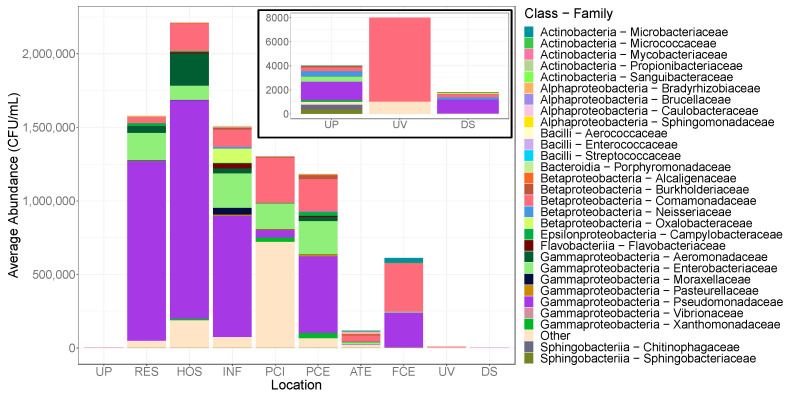
CFU/mL normalized counts of 16S rRNA gene relative abundance data for antibiotic-resistant communities at each sampling location. Combined Mallard and Sugar Creek samples at each sampling location including combined antibiotic-amended cultured communities. Relative abundance determined through 16S rRNA gene sequencing was multiplied by CFU/mL at each location to determine estimated taxonomic abundances. The inset shows the enlarged taxonomic abundances for the upstream, UV-treated, and downstream locations. Families making up <1% of the total community at each site were excluded. UP, upstream; RES, residential sewage; HOS, hospital sewage; INF, sewage influent; PCI, primary clarification influent; PCE, primary clarification effluent; ATE, aeration tank effluent; FCE, final clarification effluent; UV, UV treated effluent; DS, downstream.

Corresponding to this decrease in the viable colonies observed in ATE samples, we also saw a decrease specifically in antibiotic-resistant *Enterobacteriaceae* in the post-treatment cultures, where there is a decrease from approximately 15.7% of the pre-aeration community to 8.53% of the post-ATE community and reaching less than 1% in the UV-treated community (Figure 3, Supplementary Table S5).

As observed with heterotrophic plate counts, UV treatment greatly decreased the abundance and diversity of bacterial taxa (Figure 3). The UV-treated communities displayed the lowest Shannon alpha diversity (Supplementary Figure S2; Table S6); and with this decrease in taxonomic abundance, the cultivable resistant community was dominated by *Comamonadaceae* (Figure 3). This study reveals bacterial community changes through the treatment process and that antibiotic-resistant bacteria are significantly depleted during treatment, resulting in effluent and downstream waters resembling that of natural stream communities.

### 2.4. Net Community Changes between Upstream and Downstream Locations Are Subtle

When considering the impact of treated water release on streams, the net overall change between upstream and downstream sites is of interest, irrespective of what taxa are introduced in the wastewater treatment plant as raw sewage and removed during treatment. No significant difference was observed in total heterotrophic or antibiotic resistance counts observed between water located upstream and downstream of the treated effluent discharge point (Supplementary Table S1). Of all the sampling locations in this study, downstream water exhibited the lowest levels of resistance to the antibiotics selected (Supplementary Table S4). Due to low biomass recovery and DNA yields, downstream samples cultured on doxycycline and sulfamethoxazole could not be sequenced for taxonomic community analysis.

Multi-dimensional scaling based on Bray–Curtis distances at the genus level indicated no marked differences in beta diversity between upstream and downstream waters (Supplementary Figure S3). Community analysis revealed little overall difference in the bacteria found among upstream and downstream samples. Both up and downstream antibiotic-free communities were predominantly composed of *Pseudomonadaceae*, *Comamonadaceae*, and *Sphingomonadaceae* (Supplementary Figure S4a). The majority of the antibiotic-resistant taxa found in upstream waters were absent downstream with only *Pseudomonadaceae* and *Comamonadaceae* dominating (Figure 3). Analysis of alpha diversity did not indicate a significant difference in total sample diversity or within individual antibiotic treatments (Supplementary Table S2). These findings indicate that the treatment of wastewater and the subsequent release back into stream environments has little effect on the total cultivable communities.

### 2.5. Few Differences Are Observed between Hospital and Residential Sewage ARB Communities

The data collected afforded the opportunity to compare the composition of influent sewage taken from a purely residential trunk line to sewage taken from a trunk line in a neighborhood with a hospital. Plate counting techniques indicated significant differences in antibiotic-free and ARB colony abundances were observed between the raw sewage lines from the hospital and residential areas. Antibiotic-free growth from hospital sewage samples was three-fold higher than that found in the residential sewage (*p* = 0.001; Figure 2; Supplementary Tables S1 and S4). The combined growth of antibiotic-resistant organisms was also greater in hospital sewage samples (*p* = 0.004).

Microbial community analysis indicated no significant differences among taxa within the residential and hospital sewage sources in total heterotrophic growth or antibiotic-resistant bacteria (Supplementary Figure S5). The antibiotic-free hospital sewage community was primarily composed of *Pseudomonadaceae*, *Comamonadaceae*, and *Shewanellaceae* while 28.4% of the residential community consisted of rare and unidentified families (Supplementary Figure S4b). No significant differences in alpha diversity were observed among the two sewage communities cultivated in the presence or absence of any antibiotics utilized in this study (*p* > 0.05; Supplementary Table S2). The ARB communities recovered from both the residential and hospital sewage were primarily composed of *Pseudomonadaceae* (Figure 3). Resistant *Enterobacteriaceae*, *Aeromonadaceae*, and *Comamonadaceae* were also observed in both locations.

### 2.6. Microbial Growth in the Presence of Antibiotics

In a related paper by Lambirth et al., we determined concentrations of 10 common antibiotics in the same environmental samples used in this study. In that manuscript, we noted a significant net increase in most antibiotics from surface water downstream of the wastewater treatment plant and elevated concentrations of most antibiotics within the treatment plant environment. For this study, we chose four antibiotic compounds at relevant concentrations to determine the effect of ambient antibiotic concentrations on heterotrophic plate counts. A significant difference in bacterial counts was observed between the antibiotic-free control and each of the antibiotic amended samples (*p* < 0.001). Growth on ampicillin was high in all water samples and significantly greater than any of the other three antibiotics tested (*p* < 0.001). No count differences were observed between growth on ciprofloxacin, doxycycline, or sulfamethoxazole (Supplementary Table S1). Corresponding to the higher bacterial abundance, the antibiotic-free community diversity was significantly greater than the diversity among all of the antibiotic treatments (Supplementary Tables S2 and S6).

**Figure 4:**
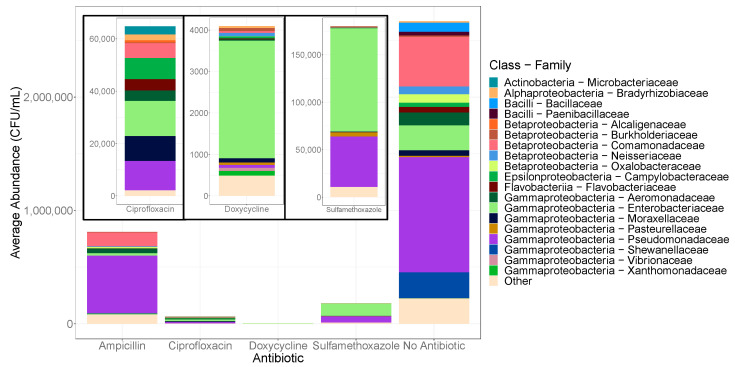
CFU/mL normalized counts from relative abundance data of the taxonomic families from each cultured antibiotic treatment. The average colony counts for each location were normalized to CFUs per ml and adjusted to the calculated relative abundance determined through 16s rRNA sequences to obtain approximated absolute abundances. Families making up <1% of the total community at each site were excluded.

*Bacillaceae* (*p* < 0.001), *Oxalobacteraceae* (*p* < 0.001), and *Moraxellaceae* (*p* < 0.001) were found to be significantly more abundant when cultured in the absence of antibiotics (Figure 4). Members of the *Bacillaceae* family were found almost exclusively in the absence of antibiotics suggesting that they are susceptible to all of the antibiotics utilized (Supplementary Table S7).

Across all sample types, *Enterobacteriaceae* and *Pseudomonadaceae* were among the core microbiome (Figure 4). Rank abundance analysis revealed that *Enterobacteriaceae* were among the top five families present under all antibiotic and control conditions and made up over half of the communities grown on sulfamethoxazole and doxycycline plates. OTUs belonging to the family *Pseudomonadaceae* were more abundant in the antibiotic-free cultivable communities, but also dominated the community grown on ampicillin (Figure 4).

The sulfamethoxazole treated communities were primarily made up of *Enterobacteriaceae* and *Pseudomonadaceae* (Figure 4). OTUs belonging to *Pasteurellaceae* were found in the highest concentration in the presence of sulfamethoxazole relative to the other antibiotic treatments (*p* < 0.001). As the lowest total growth was measured with doxycycline culturing, the abundance of all taxonomic families grown on doxycycline made up only fractions of that observed with other amendments and was predominantly composed of *Enterobacteriaceae* (67.4%).

Among the bacteria isolated with ciprofloxacin, Gammaproteobacteria were abundant at the class level (60.4%); however, no family was particularly dominant with a fairly uniform distribution among *Enterobacteriaceae*, *Pseudomonadaceae*, and *Moraxellaceae* (Figure 4). The selective conditions of the ciprofloxacin-amended agar allowed for the increased growth of several distinct taxa, including *Microbacteriaceae*, *Flavobacteriaceae*, and *Campylobacteraceae*, found in negligible concentrations with other antibiotics. *Campylobacteraceae* growth was significantly higher on ciprofloxacin (*p* < 0.001); and though classified as a rare taxon, *Microbacteriaceae* were found predominantly with ciprofloxacin amendment, in greater numbers than even the control communities.

### 2.7. Temperature Affects Bacterial Counts While Growth Medium Has a Greater Effect on Diversity

One factor in the culturability of microbes is the ability to replicate necessary growing conditions. While the focus of this study was not an exhaustive exploration of culturing conditions, we were able to sample two common growth media (LB and R2A agar) under two temperature conditions to determine the impact of culture conditions on plate count results.

Among the incubation condition combinations, the temperature had a greater effect on bacterial counts. More growth was observed at room temperature than at body temperature for both total heterotrophic colonies and ARB (*p* < 0.001; Supplementary Table S8). Media type had no significant effect on colony enumeration of total resistant organisms, though R2A agar produced more growth than LB agar. The total heterotrophic growth did appear to be influenced by the growth media (*p* = 0.048), with R2A agar yields two-fold higher than LB.

**Figure 5:**
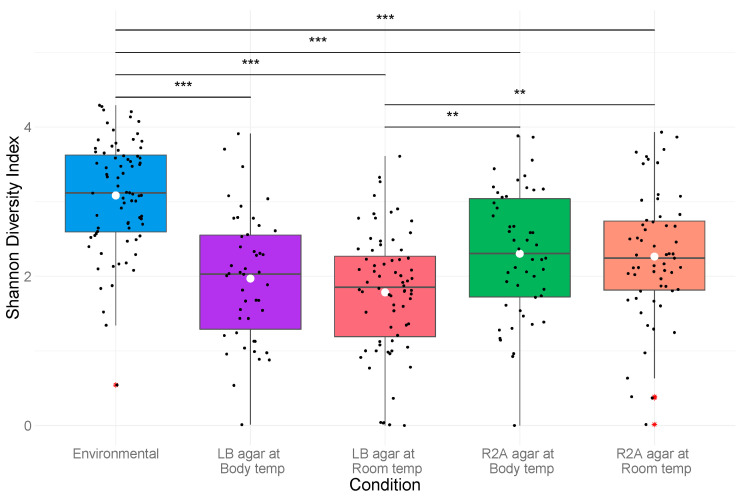
Average OTU level Shannon diversity for the culture-independent and each culture-dependent community. Significant differences are indicated with bars between the locations with statistically differential diversity values. The statistical mean is represented by a white circle. “**” indicates a *p* value of 0.001–0.01; “***” indicates a *p* value < 0.001.

In contrast to the quantitative plate counts, the media type produced a greater change in taxonomy and diversity than did temperature. No significant change in alpha diversity was observed among communities in relation to the incubation temperatures (Figure 5). Media, however, had a significant effect on antibiotic-resistant (*p* = 0.001) and total heterotrophic community diversity (*p* < 0.001). The lower nutrient concentrations of R2A agar appeared to promote the cultivation of more diverse communities relative to the nutrient-rich LB agar (Supplementary Table S9).

**Figure 6:**
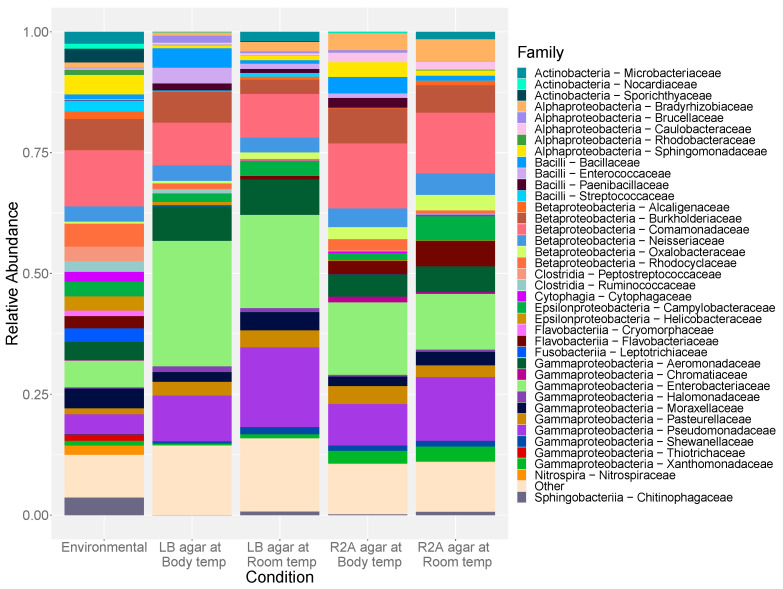
Relative abundance of the taxonomic families for the culture-independent samples and each culture-dependent condition. Families making up <1% of the total community at each site were excluded.

Between the two incubation temperatures, *Arcobacter* sp. (*Campylobacteraceae*; *p* = 0.001) and *Pseudomonas* sp. (*Pseudomonadaceae*; *p* < 0.001) were seen in greater abundance at room temperature (Figure 6). Members of the *Bacillaceae* family, particularly *Bacillus* sp., made up greater proportions of the cultured communities at 37 °C (*p* = 0.024; Supplementary Table S10). Corresponding to significant changes in diversity between the two media sources, several taxonomic families were found to be significantly more abundant on R2A agar. These families include individuals within each of the four proteobacterial classes recovered, such as *Bosea* sp. (*p* = 0.004), *Polynucleobacter* sp. (*p* = 0.024) *Arcobacter* sp. (*p* = 0.001), and *Stenotrophomonas* sp. (*p* = 0.025). Only antibiotic-resistant *Enterobacteriaceae* were found to be significantly more abundant on the LB agar (*p* = 0.013).

### 2.8. Cultivable OTUs Represent Sizeable Portions of the Total Community

Only fractions of total microbial species are routinely culturable from environmental samples. A comparison of 16S amplicon sequencing data, identified at 100% sequence similarity to the V6 region, from environmental and cultured samples allowed us to determine the proportion of culturable taxa under different combinations of conditions.

**Figure 7:**
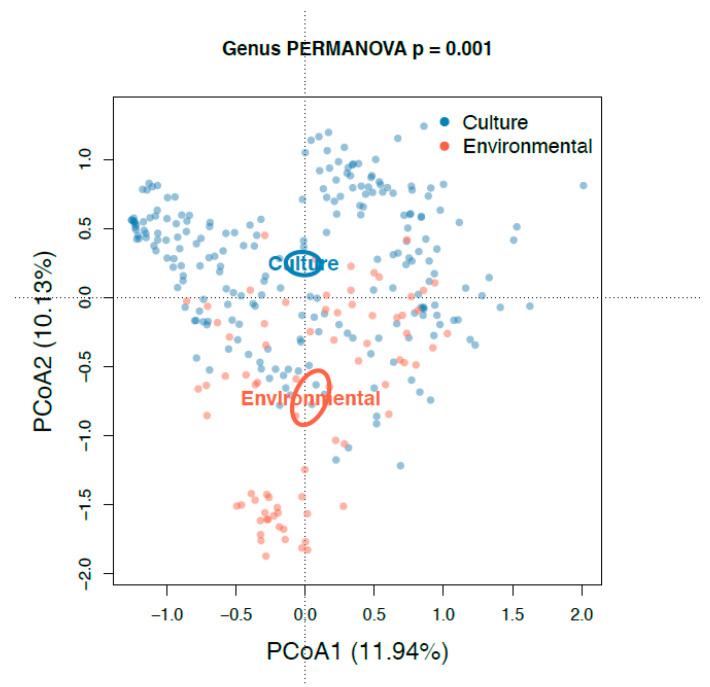
Bray Curtis PCoA ordination for all culture-independent and culture-dependent samples. Beta diversities at the genus level from 16S rRNA gene sequencing are shown with PC1 and PC2 components. Data are clustered and colored by the two sequencing approaches utilized.

**Figure 8:**
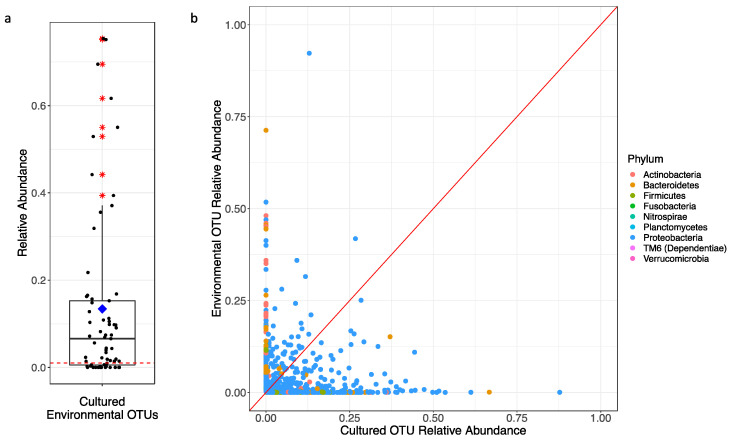
(**a**) Boxplot representing the relative abundance of OTUs also found among the culturable communities from each environmental sample. Outliers are indicated by “*” and the mean relative abundance for all samples is indicated by “♦” where the average abundance was 13.4%. (**b**) Relative abundances each culturable OTU between environmental and cultured communities. Each OTU is assigned a color based on the phyla that OTU was assigned to. The red line is the identity line representing equal abundance between sequencing approaches (Environmental = Cultured).

The principal coordinate analysis (PCoA) plot generated using Bray–Curtis dissimilarity distances showed that the 76 environmental samples were clustered apart from the 227 cultivated samples at the genus level (Figure 7). Alpha diversity of the cultured communities was significantly lower than that found in the communities of the culture-independent approach (*p* < 0.001; Figure 5). Of the 731 OTUs found in both datasets, these culturable OTUs represented an average of 13.4% of the environmental source communities across all samples (Figure 8a), while the remaining 118 OTUs unique to environmental samples made up the rest of the community. Some environmental samples were made up of culturable OTUs amounting to as high as 75% of that sample’s community.

However, the relative abundances of these OTUs found during culturing do not represent the same proportions found in the environment (Figure 8b). OTUs assigned to dominant bacterial families from the cultured communities, such as *Pseudomonadaceae* and *Enterobacteriaceae*, often represented less than 5% of the environmental communities from which they were collected (Figure 6; Supplementary Table S11). Though there was no particularly dominant family, *Comamonadaceae* (18.97%) and *Sporichthyaceae* (7.90%) were most abundant in the culture-independent samples. *Thiotrichaceae* (7.54%), *Neisseriaceae* (6.63%), and *Cytophagaceae* (5.99%) were also common community members in all environmental samples, whereas they are a minor fraction of that found upon cultivation (Supplementary Table S11). In general, OTUs within phyla Firmicutes, Actinobacteria, and Bacteroidetes were significantly less abundant in the cultured communities than within the corresponding source microbial community (*p* < 0.001), while Gammaproteobacteria were greatly enriched with cultivation (*p* < 0.001).

## 3. Discussion

This study of two Charlotte, NC WWTPs and their associated waterways indicates three major observations in regard to cultivable bacterial community changes and antibiotic resistance levels. First, community analysis reveals bacterial community changes through the treatment process where cultivable ARB are significantly depleted during treatment resulting in effluent and downstream waters resembling that of natural stream communities. Second, communities showed significant variation from total cultivable heterotrophic growth in the presence of different antibiotics, indicating a degree of resistance in these communities to particularly high antibiotic concentrations. Finally, a clear cultivability bias was observed, though not to the extent widely accepted throughout literature.

### 3.1. ARB Were Significantly Depleted during Wastewater Treatment

Water located downstream from treated effluent sites displayed lower concentrations of cultivable heterotrophic ARB than even that of the equivalent water located upstream from treatment discharge. Antibiotic resistance counts were highest among raw and pre-aeration treatment sewage. As no active biological treatment occurs during primary treatment, no significant changes in taxonomic abundance were observed at the family level among the raw (RES, HOS, INF) and primary treated (PCI, PCE) wastewater. This agrees with the general observation that the treatment process significantly reduces total numbers of resistant bacteria and supports the findings of Lambirth et al. in regard to total environmental bacteria and the presence of resistance gene markers in these sampling locations [6,8,28]. Though ARGs were found to be slightly more abundant in the downstream waters, only 9 of the 600 unique ARGs and MGEs were observed in higher concentrations downstream relative to upstream concentrations [29].

Overall, organisms associated with the human microbiome were found in greater concentrations in the sewage and wastewater samples compared to the stream samples. The treatment process was found to reduce the abundance of the human-associated microbes, specifically those classified as Bacteroidetes and Firmicutes, both in the presence and in the absence of high antibiotic concentrations. The activated sludge process reduced the total abundance of Firmicutes, while Bacteroidetes showed a decline upon UV treatment. Both of these phases of treatment are designed for microbial reduction and such reductions were observed in the heterotrophic bacterial counts [6].

Both stream communities are characteristic of typical freshwater stream communities composed primarily of Proteobacteria. Though not statistically relevant, a greater abundance of OTUs assigned as Bacteroidetes (10.2%) were observed in the downstream waters relative to those upstream (8.10%), which is consistent with the introduction of human fecal matter from the treated wastewater [30]. Overall, Charlotte Water’s treatment process appears to be effective in removing resistant bacteria with significant reductions in ARG concentrations upon activated sludge and UV treatments, though elevated concentrations of the antibiotic compounds, themselves, were observed in the surface water downstream of the treatment plants [29].

### 3.2. Multiple Phylogenetic Groups Were Resistant to at Least One Antibiotic

Bacteria classified as Gammaproteobacteria, particularly those assigned to the families *Pseudomonadaceae* and *Enterobacteriaceae*, and Betaproteobacteria showed resistance to all antibiotics utilized. Resistant communities were found in much greater abundance among raw sewage and the pre-aeration stages of treatment within the WWTP. The highest antibiotic resistance counts were observed with ampicillin. Resistance to this β-lactamase drug, developed in the 1960s, was identified as early as 1972 and is now widespread [31,32]. These ARB were dominated by Gammaproteobacteria (81.3%), of which *Pseudomonas* sp. (62.9%) and *Aeromonas* sp. (4.79%) were the most abundant with the vast majority of this growth occurring with room temperature incubations (Supplementary Figure S6). Previous findings have indicated that these two genera are almost completely resistant to this drug [33]. The *Pseudomonadaceae* family has been described as carrying several antibiotic resistance determinants along with the ability to grow on standard culture media [33,34]. Members of *Aeromonas* sp. are known to produce inducible, chromosomally encoded β-lactamases conveying resistance to some β-lactams, including ampicillin [34]. The *Aeromonadaceae* family has also been reported to harbor a wide variety of antibiotic resistance mechanisms and to acquire resistance determinants under selective pressures [35].

The antibiotic-resistant bacterial counts with sulfamethoxazole were especially dominated by Gammaproteobacteria (97.0%). ARB recovered with this drug included *Enterobacteriaceae* and *Pseudomonadaceae* across all culturing conditions with the exception of low recoveries of the latter in low-nutrient, high-temperature incubations (Figure 4 and Figure S6). This bacteriostatic antibiotic, typically used in combination with trimethoprim, has been employed for the treatment of infections in the urinary, gastrointestinal, and respiratory tracts [36]. Resistance to sulfamethoxazole has been on the rise since the late twentieth century [37]. Plasmid-encoded resistance to both sulfamethoxazole and trimethoprim can be easily transferred among these Gram-negative bacterial families resulting in increased levels of resistance to these drugs [37].

Several rare families were observed in greater relative abundance in the presence of ciprofloxacin (Figure 4). *Flavobacteriaceae* and *Campylobacteraceae* made up 6.60% and 12.1% of the ciprofloxacin-resistant growth, respectively (Supplementary Table S7). *Bradyrhizobiaceae*, *Caulobacteraceae*, *Microbacteriaceae*, *Comamonadaceae*, and *Alcaligenaceae* were also among isolates grown with this broad-range fluoroquinolone, in which R2A agar at lower incubation temperatures yielded greater taxonomic diversity. Plasmid-mediated quinolone resistance (PMQR) genes are considered the primary route for the spread of fluoroquinolone resistance [38]. These PMQR genes are often associated with mobile genetic elements responsible for multi-drug resistance (MDR) [39]. Such MDR genes encode for resistance to drug classes including quinolones, β-lactams, tetracyclines, and sulfonamides promoting the transfer of resistance across a diverse range of bacteria [39]. Nearly 70% of ciprofloxacin remains undegraded during wastewater treatment due to its recalcitrance and even non-quinolone antibiotics can increase the selective pressures favoring the persistence of PMQR genes [40,41].

The lowest antibiotic resistance numbers were observed with doxycycline. This semisynthetic tetracycline originally overcame the resistance issues of its tetracycline predecessors due to chemical modifications to improve its antimicrobial potency and spectrum [42,43]. However, over time, the utility of this drug has narrowed as the products of tetracycline-resistance genes, including Tet(A), Tet(B), and Tet(K) efflux pumps, began to recognize the compound [44]. Members of *Pasteurellaceae* and *Xanthomonadaceae* were observed at room temperature with R2A agar (Supplementary Figure S6), though *Enterobacteriaceae* were predominantly the most abundant family in all culturing conditions isolated with this tetracycline, which is not surprising considering the first description of tetracycline resistance attributed to a mutation in the drug target was observed in a *Klebsiella pneumoniae* strain [45].

In general, multiple phylogenetic groups showed high levels of resistance to at least one antibiotic. Whether it be acquired or intrinsic resistance, certain cultivable taxa were capable of withstanding these concentrations during the treatment process. As not all of the microbial community is cultivable, observing such resistant communities at these drug concentrations only shows us the small portion from the total population that is capable of being cultured. The bias observed in cultured communities compared to culture-independent methods may not be an accurate reflection of resistant community shifts as factors, irrespective of antibiotic amendment, pose selective pressures on the abundance of different phylogenetic groups. Despite this bias, community shifts paralleled those observed by Lambirth et al. in that the treated wastewater more closely resembled freshwater communities due to the efficacy of the processes used in treatment.

### 3.3. Cultivability Bias Was Apparent, But Less Severe Than Expected

Temperature had a greater effect on the number of resistant colonies observed, while media appeared to have little consequence on bacterial quantity (Supplementary Table S1). However, the media did have a significant influence on which organisms within the population were capable of growth (Figure 6). The quantitative findings are consistent with observations summarized by Allen et al. (2004) in that lower incubation temperatures favor the growth of water-based microbes. Though not statistically meaningful, R2A agar resulted in higher yields, likely promoted by the low-nutrient, low-ionic strength formulation of this medium [46].

The diversity of the culture-dependent communities was significantly lower than that of the culture-independent approach (Figure 5). While none of the culturing conditions resulted in growth reflective of such highly diverse communities, sequence reads of the cultivable OTUs were observed in the culture-independent bacterial communities in higher concentrations than anticipated. OTUs found within the cultured bacterial communities made up, on average, 13.4% of the total OTUs recovered with the culture-independent methodology. These common, culturable OTUs made up greater proportions of the total communities than the commonly accepted 1% culturability paradigm. This widely accepted paradigm, likely conceived as early as 1995 [47], is based on the concept of “the great plate count anomaly” proposed by Staley and Konopka (1985).

According to a literature search conducted by Adam Martiny (2019), there are several interpretations of this axiom with current explanations being that only 1% of the cells or taxa in a community can be cultivated, despite the use of all available culturing methods. It is likely, though, that the original interpretation was that only 1% of the cells in a community can be cultured on standard agar medium [48]. At the time this paradigm was originally conceived, only about 5000 bacterial species had been described [47]. Since that time, efforts in cultivating abundant bacteria and culturing methodology innovations have allowed for the growth of a greater proportion of bacterial communities, and, as of 2017, over 15,000 species have been described [49]. As evidenced by the current findings and that of other such studies, this standard of thinking is no longer valid. Several other studies have also indicated culturable bacterial yields of 70% or more in several diverse environments [18,50].

Though higher culturable yields were observed, the proportions of these shared OTUs within each sample were not always reflective of those found in the microbial source material (Figure 8b). The sequences obtained from the culture-dependent approach were dominated by Gammaproteobacteria (66.1%), of which *Enterobacteriaceae*, *Pseudomonadaceae*, and *Aeromonadaceae* were among the most abundant families. These families made up significantly greater proportions of the cultured communities than they did in the source communities, which were primarily composed of Betaproteobacteria (38.6%). Selection of Gammaproteobacteria has been described as a cultivation bias in aquatic bacterial communities, likely as the result of specific life strategies employed by members of this class [51]. These findings are supported in a similar study, by Garcia-Armisen et al. (2013), that observed analogous results in regard to community compositions of culture-dependent and -independent samples [35]. In general, higher concentrations of OTUs were found to be culturable than previously thought; though the proportions of these OTUs within their respective communities varied from those identified using culture-independent approaches.

## 4. Materials and Methods

### 4.1. Sampling Locations

We investigated two water systems associated with the wastewater treatment process: the upstream and downstream surface water associated with the treated effluent discharge point, and raw and subsequently treated sewage sampled at several sites inside the treatment plant. Charlotte has no large body of water within the city itself but does have a network of small creeks and streams, which are important ecological systems that are often features of public greenways and park facilities. The regional water utility, Charlotte Water, operates five major wastewater treatment facilities, from which we selected Mallard Creek Water Reclamation and Sugar Creek Wastewater Treatment Plant. The Mallard Creek facility is estimated to process 12 million gallons daily (MGD) with effluent release into Mallard Creek, part of the Yadkin-Pee Dee river basin. The Sugar Creek facility is rated for 20 MGD and discharges into Little Sugar Creek, which becomes Sugar Creek before eventually joining the Catawba river basin. Both WWTPs are activated biosolids plants that use physical screens, grit removal stations, primary clarifiers with subsequent processing of activated biosolids of anoxic and aerobic zones, and secondary clarification ending with ultraviolet disinfection. Additional details related to specific parameters can be found in Lambirth et al. (2018).

### 4.2. Selection of Sampling Sites

Sampling locations were selected from sewer lines routed to each facility, multiple sites within each WWTP, water upstream from effluent release, and the downstream receiving watershed in April 2016 (Figure 1). Composite samples from hospital (HOS) and residential (RES) sewage lines to each WWTP were sampled prior to the merging of the two lines into a main sewer line. The sites selected within the Mallard Creek facility included the collective raw influent (INF), the primary clarifier influent (PCI) and primary clarifier effluent (PCE) which has undergone primary treatment, and the activated sludge processed aeration tank effluent (ATE) and final clarifier effluent (FCE). Analogous sampling sites were chosen at the Sugar Creek facility, including INF, PCE, ATE, and FCE. The location of sampling points at the Sugar Creek plant did not allow for the collection of a PCI sample but did allow for access to the ultraviolet disinfected effluent (UV), which was not accessible at the Mallard Creek facility. Stream sites upstream (UP) and downstream (DS) of each plant’s effluent pipe were selected to assess changes in the stream microbiome due to effluent release.

### 4.3. Sample Collection

Stream samples were manually collected by submerging sterile 1 L Nalgene bottles several inches below the surface water with the bottle mouth oriented against the streamflow. Composite samples of the raw and treated sewage throughout the treatment plant were collected via peristaltic ISCO 6712 auto-samplers (Teledyne, Lincoln, NE, USA) pulling 150 mL every 30 min over a 24-h period into sterile 2.5-gallon carboys on ice. After collection, sewage samples were transferred from their respective carboys into sterile 1 L Nalgene bottles through a peristaltic pump within a sterile biosafety cabinet with replacement pump tubing in between each sample transfer. All samples were stored at 4 °C prior to sample analysis.

### 4.4. Environmental DNA Extraction

Each water sample was vacuum-filtered onto 0.45-micron cellulose filters (MoBio, Carlsbad, CA, USA) in 100 mL aliquots until sample flow ceased for extraction of total genomic DNA. Filter material from these environmental samples was aseptically removed from the vacuum manifold and cut into strips, DNA was extracted from the filter residue using FastDNA^®^ SPIN Kit for Soil (MP Biomedicals, Solon, OH, USA) according to the manufacturer’s instructions. DNA quantification was performed using a Qubit^®^ 2.0 Fluorometer (Thermo Fisher Scientific Inc., Waltham, MA, USA) and Nanodrop™ (Thermo Fisher Scientific Inc.). All isolated DNA was stored at −80 °C until sequencing was performed.

### 4.5. Heterotrophic Plate Counts

Antibiotics were amended to nutrient-rich LB (containing 10 g NaCl per liter) and nutrient-poor R2A agar to evaluate bacterial resistance compared to total heterotrophic growth on antibiotic-free agar. The following frequently prescribed antibiotics were added to the culture media in higher concentrations than are typically prescribed: ampicillin, 1000 μg/mL; ciprofloxacin, 50 μg/mL; doxycycline, 100 μg/mL; sulfamethoxazole, 1000 μg/mL. Each of these antibiotics represent a major antibiotic class and mechanism of action: penicillins (ampicillin) with peptidoglycan layer formation inhibition, DNA gyrase inhibition by fluoroquinolones (ciprofloxacin), protein formation inhibiting tetracyclines (doxycycline), and sulfonamides (sulfamethoxazole) inhibiting folate synthesis. All growth media were sterilized by autoclaving (30 min; 121 °C) prior to the addition of sterile-filtered antibiotic stocks.

Water samples were serially diluted 10-fold in sterile deionized (DI) water and applied, in triplicate, to agar plates each amended with one antibiotic and to antibiotic-free controls using standard spread plating techniques. Both antibiotic-amended and control R2A and LB plates were incubated at room temperature for 5 to 7 days (21.5 °C) and at human body temperature (37 °C) for 2 to 3 days. Upon completion of the incubation period, colony-forming units (CFU) were visually quantified and statistical comparisons were performed.

### 4.6. Culture DNA Extractions

All biomass from the cultured heterotrophic bacteria was aseptically collected for DNA extraction into sterile 1.5 mL microcentrifuge tubes. Biomass from the replicates was pooled and DNA from each pooled sample was extracted using the UltraClean^®^ Microbial DNA Isolate Kit (MoBio) according to the manufacturer’s instructions and quantified using a Qubit^®^ 2.0 Fluorometer. Isolated DNA was stored at −20 °C per manufacturer’s recommendation.

### 4.7. 16S rRNA Library Preparation and Illumina Sequencing

All sequencing was performed at the David H. Murdock Research Institute. Ribosomal amplicon libraries were created from collected DNA templates using universal primers of the V6 variable region of the 16S rRNA gene (967f forward primer, 5′-CAACGCGARGAACCTTACC-3′; 1061r reverse primer, 5′-ACAACACGAGCTGACGAC-3′). Each sample was uniquely indexed and sequenced with 125 bp paired-end reads on an Illumina^®^ HiSeq 2500 flow cell. All raw 16S rRNA sequences have been submitted to the National Center for Biotechnology Information (NCBI) under BioProject ID number PRJNA657079.

### 4.8. 16S Sequencing Analysis Using QIIME2

The Quantitative Insights Into Microbial Ecology (QIIME2; v. 2019.1) pipeline was used for 16S community analysis of all samples [52]. Forward and reverse sequence reads were paired, denoised, and quality trimmed to 115 base pairs using DADA2 to generate amplicon sequence variants [53]. Amplicon sequence variants (ASVs) with a frequency less than ten were filtered out. Open-reference OTU clustering was performed with a sequence identity threshold of 100% using the SILVA 128 database [54,55]. De novo chimera detection was performed using the UCHIME algorithm and chimeric and borderline chimeric sequences were filtered out with VSearch [56,57]. All singleton OTUs were removed using the QIIME2 “feature-table filter” function. The resulting OTU table was again filtered using the same filtering function to remove all OTUs with a relative abundance of <0.01% across all samples to eliminate rare taxa and samples with fewer than 10,000 sequence reads. This OTU table was then used for subsequent analyses.

Consensus sequence taxonomy classification was performed with VSearch using the SILVA 128 16S rRNA gene sequence and taxonomic reference base. Finally, OTUs were summarized at a minimum relative abundance of 3% in each sample at the family level and all low abundance and unclassified OTUs were reclassified as “Other” [58].

### 4.9. Statistical Methods

Analysis of culture and environmental datasets were analyzed using the automated BioLockJ pipeline (https://github.com/BioLockJ-Dev-Team/BioLockJ (accessed on 23 March 2021)). By combining the CFU count data and the relative abundance of observed taxa, we were able to estimate the absolute abundance of taxa within the samples. Relative abundance, determined through 16S rRNA gene sequencing, was multiplied by the calculated CFU/mL to determine estimated taxonomic abundances. This estimated abundance should not be misconstrued as the definitive absolute abundance of the organisms found on each culturing plate as not all organisms carry the same copy numbers of the 16S rRNA gene [59]. Each dataset was normalized according to the following formula to account for differences in sequencing depth among all samples:

(1) $$\log_{10}\left(\left(\frac{Raw\;sequence\;count\;in\;sample}{\#\;of\;sequences\;in\;sample}\;\times \;Average\;\#of\;sequences\;per\;sample\right)\;+\;1\right)$$

As replicate samples clustered closely together, the sample with the deepest sequencing was retained as a representative for that measurement. Taxa present in less than 10% of all samples were removed to avoid the detection of stochastic differences in rare taxa and to preserve power by not requiring multiple hypothesis testing for these rare taxa.

Statistical comparisons in microbial abundance were performed using linear regression models via the “lm” function in R to evaluate significant differences between taxa isolated from each treatment stage and culturing condition using the log10 normalized taxa counts [60]. The “summary” function was used to generate taxonomic *p* values for each linear model. The resulting *p* values were corrected for multiple hypothesis testing with the Benjamini–Hochberg procedure and were considered significant when the False Discovery Rate was <5% [61].

Diversity analyses were performed with the QIIME2 “diversity alpha” function where the Shannon Diversity Index (SDI) and observed OTUs were calculated for each sample. Linear models were constructed to compare Shannon diversity and log10 normalized colony counts (with an added pseudocount) across all variables (SDI~Site + Location + Temperature + Media + Antibiotic). Beta diversity was determined using Bray–Curtis equilibrium distances and plotted using the Constrained Analysis of Principal Coordinates (CAP) ordination method via the “capscale” function in the Vegan package in R [62]. PERMANOVA testing on the Bray–Curtis distances was further used to compare microbial composition between individual variables. All analyses and visualizations were generated using a Dockerized version of R Studio (Version 4.0.2) and the R scripts are available at https://github.com/asorgen/UEGP_WastewaterCulture (accessed on 23 March 2021).

## 5. Conclusions

Cultivation practices in the study of bacteria are fundamental to our knowledge of their ecological roles. Even in the era of bioinformatic advancements, to understand the extent to which microbial populations have been characterized, we must quantify the fraction of bacterial cells that share physiologies with cultured organisms. Though methodological laboratory practices are still far from successfully culturing all bacterial community members from most environments, the principle that only 1% of the microbial population in all environments are culturable is outdated [63]. It is important to note that this study only demonstrated resistance to four individual antibiotics and that many viable organisms within the sample water are not represented due to culture biases. As we tested antibiotics individually, assumptions of MDR organisms and their prevalence cannot be drawn. However, it has been commonly observed that the harboring of a single resistance gene can confer resistance to multiple antibiotics and that several such genes are often co-localized on MGEs able to undergo HGT as a unit [64,65]. In conjunction with previous work, we were able to determine that, indeed, overall ARB and the associated ARGs are significantly reduced during treatment. Despite these encouraging findings, the dissemination of ARB and ARGs are but one mechanism for the propagation of antibiotic resistance and thus further studies are required to assess the effect of released antibiotic compounds, alone, on environmental microbial communities and the potential for such resistance determinants to reach clinically relevant pathogens.

## Data Availability

All sequence data have been submitted to the National Center for Biotechnology Information (NCBI) under BioProject ID number PRJNA657079. All analyses and visualizations were generated using a Dockerized version of R Studio (Version 4.0.2) and the R scripts are available at https://github.com/asorgen/UEGP_WastewaterCulture (accessed on 23 March 2021).

## Supplementary Materials

The following are available online at https://www.mdpi.com/article/10.3390/antibiotics10040352/s1, Table S1: Differential abundance *p* values of colony counts; Table S2: Differential comparison *p* values for Shannon diversity; Table S3: Relative abundance of taxonomic families recovered from Mallard and Sugar Creek; Table S4: Average colony counts for each sampling location and antibiotic amendment; Table S5: Relative abundance of antibiotic-resistant taxonomic families from sampling locations; Table S6: Average Shannon diversity for each sampling location; Figure S1: Beta diversities at genus level between Mallard and Sugar Creek; Table S7: Average relative abundance and differential abundance results for families based on antibiotic amendment; Figure S2: Average OTU level Shannon diversity of ARB communities for all locations; Table S8: Average colony counts for incubation temperature and media; Figure S3: Beta diversity and taxonomic differences between stream communities; Figure S4: Relative abundance for stream and sewage communities; Figure S5: Beta diversity and taxonomic differences between sewage communities; Table S9: Average Shannon diversity for incubation temperatures and media; Table S10: Significant differences in taxa between incubation temperatures and media; Table S11: Relative abundance and significant differences in cultured and environmental families; Figure S6: Relative abundance of families based on incubation condition and antibiotic amendment.
