# Supplementary material for: Characterization of Environmental and Cultivable Antibiotic-Resistant Microbial Communities Associated with Wastewater Treatment
Source: Antibiotics (Basel). 2021 Mar 26;10(4):352. doi: 10.3390/antibiotics10040352 (PMC8066808; doi:10.3390/antibiotics10040352)
Supplement: Supplementary file 1 [file antibiotics-10-00352-s001.pdf]

Supplementary File

Table S1. Differential abundance *p* values of colony counts (normalized to CFU/ml) between each WWTP site, incubation temperature and media, stage of treatment, and antibiotic utilized for combined ARB, the total heterotrophic growth (HPC), and each individual antibiotic treatments.

Terms	ALL	ARB	HPC	AMP	CIP	DOX	SUL
Mallard-Sugar	0.740	0.026	<0.001	0.004	0.553	<0.001	0.518
BT-RT	<0.001	<0.001	<0.001	<0.001	<0.001	<0.001	<0.001
LB-R2A	0.048	0.545	<0.001	<0.001	0.667	0.661	<0.001
UPA-DSA	0.168	0.138	0.759	0.378	0.759	0.017	0.443
RES-HOS	0.001	0.004	0.003	<0.001	0.106	0.677	0.819
RES-INF	0.008	0.004	0.629	<0.001	0.025	0.590	0.781
HOS-INF	0.416	0.973	0.012	0.583	0.530	0.339	0.613
INF-ATE	<0.001	<0.001	0.831	<0.001	<0.001	0.001	<0.001
PCI-PCE	0.104	0.469	0.003	0.163	0.612	0.177	<0.001
PCI-ATE	<0.001	<0.001	0.029	0.003	<0.001	0.195	<0.001
PCE-ATE	<0.001	<0.001	0.312	<0.001	<0.001	0.001	<0.001
ATE-FCE	0.001	<0.001	0.594	0.089	0.842	<0.001	<0.001
ATE-UV	<0.001	<0.001	0.023	<0.001	0.056	<0.001	<0.001
FCE-UV	<0.001	<0.001	0.007	0.001	0.039	<0.001	0.897
UV-DSA	0.424	0.548	<0.001	0.958	0.334	0.925	0.897
HPC-AMP	<0.001	n/a	n/a	n/a	n/a	n/a	n/a
HPC-CIP	<0.001	n/a	n/a	n/a	n/a	n/a	n/a
HPC-DOX	<0.001	n/a	n/a	n/a	n/a	n/a	n/a
HPC-SUL	<0.001	n/a	n/a	n/a	n/a	n/a	n/a
AMP-CIP	<0.001	n/a	n/a	n/a	n/a	n/a	n/a
AMP-DOX	<0.001	n/a	n/a	n/a	n/a	n/a	n/a
AMP-SUL	<0.001	n/a	n/a	n/a	n/a	n/a	n/a
CIP-DOX	0.829	n/a	n/a	n/a	n/a	n/a	n/a
CIP-SUL	0.652	n/a	n/a	n/a	n/a	n/a	n/a
DOX-SUL	0.814	n/a	n/a	n/a	n/a	n/a	n/a

ALL, total heterotrophic (no antibiotic amendment) and antibiotic resistant; HPC, total heterotrophic growth; AMP, ampicillin; CIP, ciprofloxacin; DOX, doxycycline; SUL, sulfamethoxazole; UPA, upstream; RES, residential sewage; HOS, hospital sewage; INF, sewage influent; PCI, primary clarification influent; PCE, primary clarification effluent; ATE, aeration tank effluent; FCE, final clarification effluent; UV, UV treated effluent; DSA, downstream.

Table S2. Differential comparison *p* values for Shannon diversity between each WWTP site, incubation temperature and media, stage of treatment, and antibiotic utilized for combined ARB, the total heterotrophic growth (HPC), and each individual antibiotic treatments.

Terms	ALL	ARB	Neg	Amp	Cip	Dox	Sulf
Culture-Environmental	<0.001	n/a	n/a	n/a	n/a	n/a	n/a
Mallard-Sugar	0.643	0.209	0.478	0.145	0.138	0.428	0.623
BT-RT	0.903	0.397	0.110	0.307	0.318	0.688	0.926
LB-R2A	<0.001	0.001	0.022	<0.001	0.635	0.028	0.094
UPA-DSA	0.844	0.791	0.837	0.777	0.628	n/a	n/a
RES-HOS	0.545	0.266	0.425	0.210	0.602	0.478	0.802
RES-INF	0.635	0.170	0.077	0.025	0.224	0.585	0.793
HOS-INF	0.888	0.826	0.252	0.322	0.509	0.214	0.646
INF-ATE	0.559	0.040	0.010	0.006	0.657	0.658	0.084

PCI-PCE	0.321	0.689	0.147	0.810	0.899	0.308	0.251
PCI-ATE	0.125	0.120	0.339	0.215	0.633	0.378	0.018
PCE-ATE	0.537	0.193	0.464	0.054	0.546	0.817	0.161
FCE-UV	0.066	0.038	0.989	0.119	n/a	n/a	n/a
UV-DSA	0.011	0.050	0.077	0.044	n/a	n/a	n/a
HPC-AMP	<0.001	n/a	n/a	n/a	n/a	n/a	n/a
HPC-CIP	<0.001	n/a	n/a	n/a	n/a	n/a	n/a
HPC-DOX	<0.001	n/a	n/a	n/a	n/a	n/a	n/a
HPC-SUL	0.008	n/a	n/a	n/a	n/a	n/a	n/a
AMP-CIP	0.025	n/a	n/a	n/a	n/a	n/a	n/a
AMP-DOX	0.588	n/a	n/a	n/a	n/a	n/a	n/a
AMP-SUL	0.136	n/a	n/a	n/a	n/a	n/a	n/a
CIP-DOX	0.102	n/a	n/a	n/a	n/a	n/a	n/a
CIP-SUL	0.001	n/a	n/a	n/a	n/a	n/a	n/a
DOX-SUL	0.056	n/a	n/a	n/a	n/a	n/a	n/a

ALL, total heterotrophic (no antibiotic amendment) and antibiotic resistant; HPC, total heterotrophic growth; AMP, ampicillin; CIP, ciprofloxacin; DOX, doxycycline; SUL, sulfamethoxazole; UPA, upstream; RES, residential sewage; HOS, hospital sewage; INF, sewage influent; PCI, primary clarification influent; PCE, primary clarification effluent; ATE, aeration tank effluent; FCE, final clarification effluent; UV, UV treated effluent; DSA, downstream.

Table S3. Relative abundance of taxonomic families recovered from the Mallard and Sugar Creek WWTPs.

Phylum	Class	Family	Mallard	Sugar
Actinobacteria	Actinobacteria	*Microbacteriaceae*	2.59%	2.07%
	Flavobacteriia	*Flavobacteriaceae*	2.84%	1.78%
	Sphingobacteriia	*Chitinophagaceae*	1.21%	0%
		Sphingobacteriaceae	1.56%	<0.01%
Firmicutes	Bacilli	*Bacillaceae*	2.79%	0.30%
		Paenibacillaceae	1.23%	<0.01%
Proteobacteria	Alphaproteobacteria	*Caulobacteraceae*	2.60%	0.52%
		Bradyrhizobiaceae	2.20%	1.42%
	Betaproteobacteria	*Alcaligenaceae*	1.37%	0.01%
		Burkholderiaceae	1.87%	3.16%
		Comamonadaceae	8.14%	10.19%
		Neisseriaceae	0.64%	6.24%
	Epsilonproteobacteria	*Campylobacteraceae*	1.40%	1.53%
	Gammaproteobacteria	*Aeromonadaceae*	1.29%	5.92%
		Shewanellaceae	<0.01%	1.26%
		Enterobacteriaceae	34.03%	29.51%
		Moraxellaceae	0.86%	2.94%
		Pseudomonadaceae	17.10%	18.64%
		Xanthomonadaceae	2.03%	<0.01%
Other	Other	Other	14.24%	14.51%

Table S4. Average colony counts (normalized to CFU/ml) for each sampling location and individual antibiotic amendment.

Location	HPC	AMP	CIP	DOX	SUL	Location Avg	Location Avg (ARB only)
UPA	5.E+03	6.E+02	7.E+01	3.E+01	1.E+01	1.E+03	2.E+02
RES	7.E+05	4.E+05	2.E+04	1.E+03	7.E+04	2.E+05	1.E+05
HOS	3.E+06	6.E+05	2.E+04	1.E+03	2.E+05	8.E+05	2.E+05

INF	1.E+06	4.E+05	4.E+04	2.E+03	6.E+04	3.E+05	1.E+05
PCI	8.E+05	4.E+05	6.E+03	1.E+03	6.E+04	3.E+05	1.E+05
PCE	1.E+06	2.E+05	2.E+04	2.E+03	6.E+04	3.E+05	8.E+04
ATE	4.E+05	2.E+04	2.E+03	9.E+02	2.E+03	8.E+04	7.E+03
FCE	5.E+05	1.E+05	5.E+03	7.E+02	0.E+00	1.E+05	3.E+04
UV	1.E+05	1.E+03	0.E+00	0.E+00	0.E+00	3.E+04	3.E+02
DSA	4.E+03	3.E+02	2.E+01	3.E+00	0.E+00	8.E+02	7.E+01
Antibiotic Avg	8.E+05	2.E+05	1.E+04	9.E+02	4.E+04		

Table S5. Relative abundance of the antibiotic resistant taxonomic families recovered from all sampling locations within the wastewater treatment process.

Phylum	Class	Family	UPA	RES	HOS	INF	PCI	PCE	ATE	FCE	UV	DSA
Actinobacteria	Actinobacteria	*Microbacteriaceae*	0.43%	0.02%	<0.01%	0.05%	0%	0%	2.00%	3.37%	0%	0.84%
Bacteroidetes	Flavobacteriia	*Flavobacteriaceae*	<0.01%	0.16%	0.46%	2.41%	0.17%	0.61%	<0.01%	<0.01%	0%	0%
	Sphingobacteriia	*Chitinophagaceae*	5.05%	<0.01%	<0.01%	<0.01%	<0.01%	<0.01%	<0.01%	<0.01%	0%	<0.01%
		Sphingobacteriaceae	4.80%	0%	0%	0%	0%	0%	0%	0%	0%	0%
Proteobacteria	Alphaproteobacteria	*Bradyrhizobiaceae*	0.01%	0.48%	0.06%	0.12%	0.14%	0.01%	1.05%	0.04%	0%	<0.01%
		Brucellaceae	0%	0.05%	0%	<0.01%	0%	0%	1.48%	<0.01%	0%	0%
		Caulobacteraceae	0%	0.05%	<0.01%	0.05%	0.07%	0.14%	7.65%	0.29%	0%	0.53%
		Sphingomonadaceae	0%	<0.01%	0.10%	0.15%	<0.01%	<0.01%	<0.01%	0.03%	0%	1.23%
	Betaproteobacteria	*Alcaligenaceae*	0%	0%	0%	<0.01%	<0.01%	0.29%	1.52%	<0.01%	0%	0%
		Burkholderiaceae	4.38%	0.11%	0.06%	0.97%	0.49%	2.45%	6.88%	0.70%	0%	0.73%
		Comamonadaceae	10.28%	2.43%	8.74%	8.26%	22.51%	20.65%	43.81%	54.30%	87.49%	16.43%
		Neisseriaceae	12.95%	0.04%	<0.01%	0.89%	0.01%	0.31%	<0.01%	0.07%	0%	10.22%
		Oxalobacteraceae	0%	<0.01%	0.02%	6.79%	<0.01%	<0.01%	0.06%	0.70%	0%	0.11%
	Epsilonproteobacteria	*Campylobacteraceae*	<0.01%	1.41%	0.29%	0.24%	0.11%	1.24%	0.06%	<0.01%	0%	<0.01%
	Gammaproteobacteria	*Aeromonadaceae*	0.63%	2.76%	10.11%	2.45%	<0.01%	2.30%	3.38%	0.01%	0%	<0.01%
		Enterobacteriaceae	4.83%	11.99%	3.19%	11.97%	15.67%	15.79%	8.53%	0.23%	0.01%	<0.01%
		Moraxellaceae	<0.01%	0.12%	<0.01%	3.25%	0.09%	<0.01%	<0.01%	0.02%	0%	<0.01%
		Pseudomonadaceae	48.63%	76.98%	68.01%	56.56%	4.87%	46.15%	3.01%	39.82%	0%	69.56%
		Xanthomonadaceae	4.12%	<0.01%	0.45%	<0.01%	2.06%	3.37%	0.19%	0.07%	0%	0.10%
Other	Other	Other	3.89%	3.39%	8.49%	5.84%	53.81%	6.70%	20.39%	0.35%	12.50%	<0.01%

UPA, upstream; RES, residential sewage; HOS, hospital sewage; INF, sewage influent; PCI, primary clarification influent; PCE, primary clarification effluent; ATE, aeration tank effluent; FCE, final clarification effluent; UV, UV treated effluent; DSA, downstream.

Table S6. Average Shannon diversity value for each sampling location and individual antibiotic amendment.

Location	HPC	AMP	CIP	DOX	SUL	Location Avg	Location Avg (ARB only)
UPA	3.09	2.34	2.27	0.96	2.47	2.58	2.19
RES	2.78	1.50	1.37	1.77	2.15	1.85	1.69
HOS	2.40	2.04	1.58	2.00	2.04	2.02	1.90
INF	2.05	2.34	1.84	1.52	2.17	1.98	1.96
PCI	3.37	2.26	1.44	2.07	2.43	2.26	2.04
PCE	2.72	2.13	1.34	1.47	2.12	2.00	1.80

ATE	2.95	1.48	1.91	1.65	1.45	1.97	1.57
FCE	2.28	1.88	1.11	2.40		1.99	1.90
UV	2.11	0.82				1.59	0.82
DSA	3.04	2.08	1.76			2.64	2.01
Antibiotic Avg	2.68	1.89	1.46	1.39	1.48		

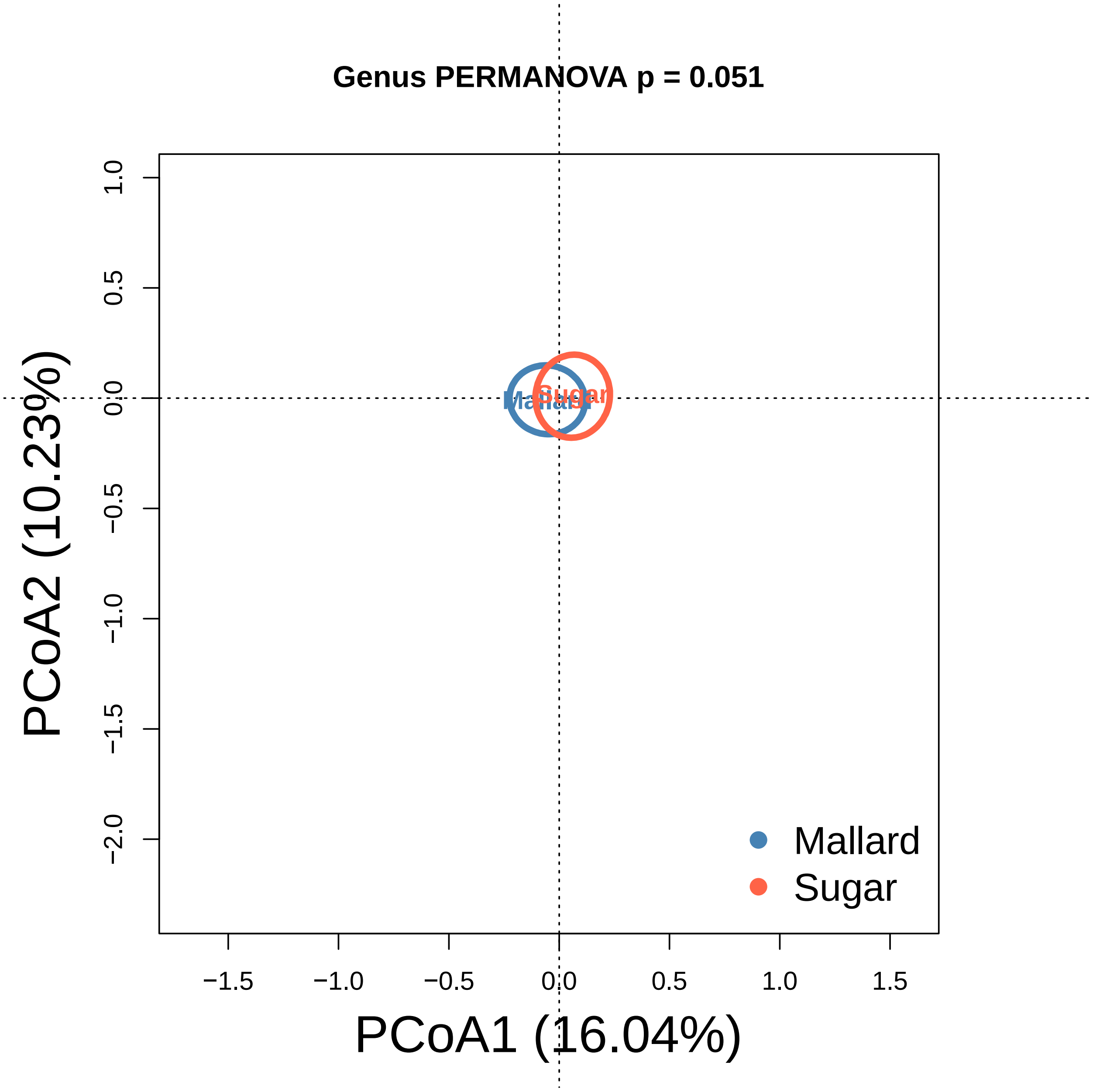

Figure S1. Beta diversities at the genus level from 16S rRNA gene sequencing. PCoA ordination for Mallard and Sugar Creek samples with PC1 and PC2 components. Data colored by the two sampling sites.

Table S7. Average relative abundance of taxonomic families in individual antibiotic treatments and associated *p* values comparing differential abundance.

Phylum	Class	Family	HPC	AMP	CIP	DOX	SUL	*p* value
Actinobacteria	Actinobacteria	*Microbacteriaceae*	<0.01%	<0.01%	4.72%	<0.01%	0%	n.s.
Bacteroidetes	Flavobacteriia	*Flavobacteriaceae*	1.88%	0.54%	6.60%	0.64%	<0.01%	<0.001
Firmicutes	Bacilli	*Bacillaceae*	2.99%	<0.01%	<0.01%	0.01%	0%	<0.001
Proteobacteria	Alphaproteobacteria	*Bradyrhizobiaceae*	0.45%	<0.01%	3.34%	1.13%	0.02%	n.s.
	Betaproteobacteria	*Alcaligenaceae*	0.06%	<0.01%	1.24%	<0.01%	<0.01%	n.s.
		Burkholderiaceae	0.42%	0.80%	0.40%	1.80%	0.56%	<0.001

		Comamonadaceae	15.90%	15.01%	8.64%	1.01%	0.24%	<0.001
		Oxalobacteraceae	2.84%	1.78%	0.01%	0.25%	<0.01%	<0.001
		Neisseriaceae	2.44%	0.29%	0.01%	1.80%	<0.01%	<0.001
	Epsilonproteobacteria	*Campylobacteraceae*	1.27%	0.02%	12.05%	1.04%	0.07%	<0.001
	Gammaproteobacteria	*Aeromonadaceae*	4.18%	4.79%	5.99%	0.74%	0.26%	<0.001
		Shewanellaceae	8.35%	0.07%	<0.01%	0.01%	0%	<0.001
		Enterobacteriaceae	8.00%	2.59%	20.24%	67.40%	59.70%	<0.001
		Pasteurellaceae	0.51%	0.04%	<0.01%	1.57%	2.65%	<0.001
		Moraxellaceae	1.70%	<0.01%	14.22%	2.30%	0.25%	<0.001
		Pseudomonadaceae	37.13%	62.92%	17.00%	1.64%	29.10%	<0.001
		Vibrionaceae	0.06%	0%	<0.01%	1.77%	0%	n.s.
		Xanthomonadaceae	0.05%	0.91%	<0.01%	2.63%	<0.01%	n.s.
Other	Other	Other	10.54%	10.22%	5.55%	14.29%	7.15%	n.s.

HPC, total heterotrophic growth; AMP, ampicillin; CIP, ciprofloxacin; DOX, doxycycline; SUL, sulfamethoxazole; n.s.– not significant

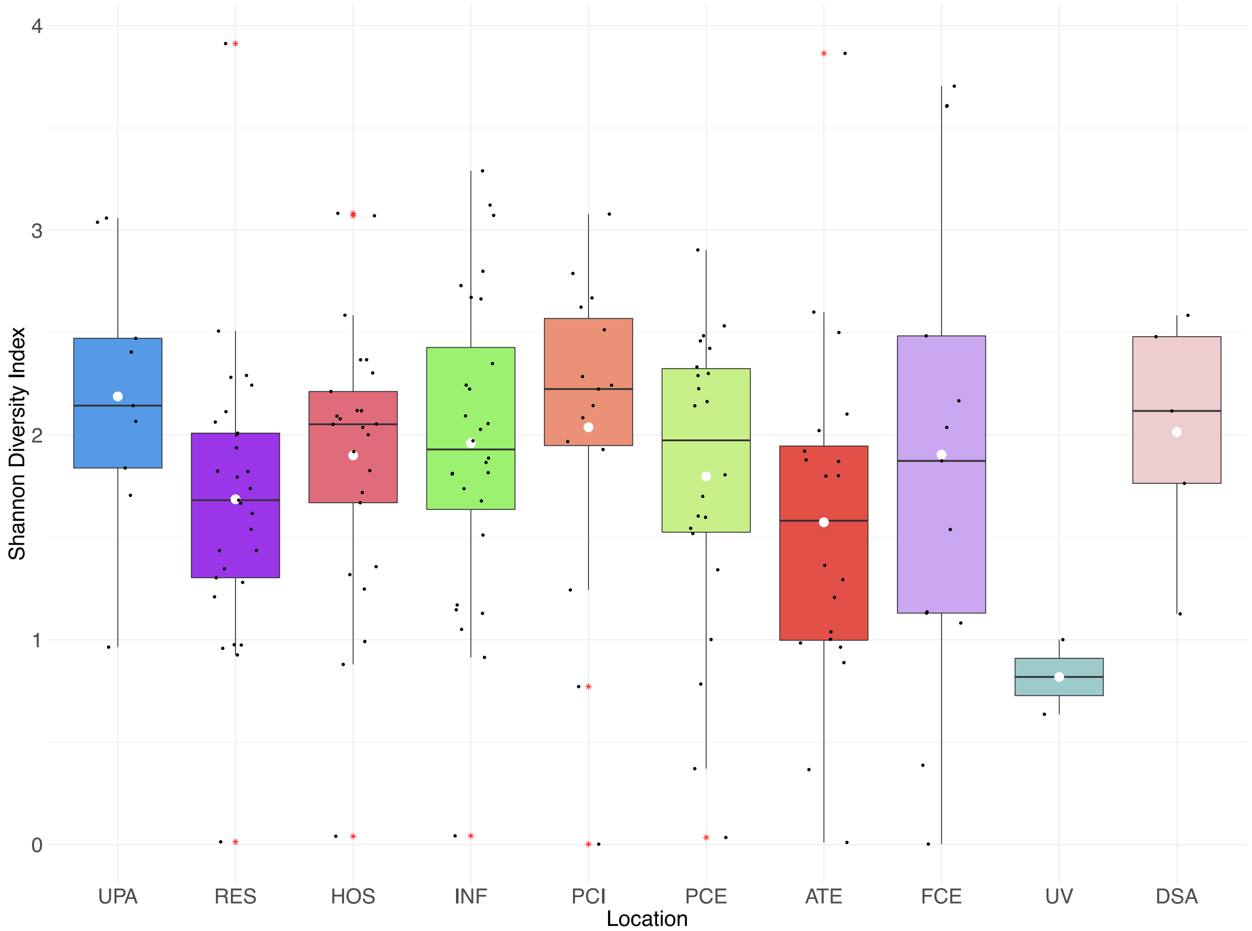

Figure S2. Average OTU level Shannon diversity of combined antibiotic resistant communities for all sampling locations. Mallard and Sugar Creek samples were combined by sampling location. Significant differences are indicated with bars between the locations with statistically differential diversity values. "*" indicates a *p* value of 0.01 – 0.05; "**" indicates a *p* value of 0.001 – 0.01; "***" indicates a *p* value <0.001. Outliers are indicated with red asterisks and the statistical mean is represented by a white circle. UPA, upstream; RES, residential sewage; HOS, hospital sewage; INF, sewage influent; PCI, primary clarification influent; PCE, primary clarification effluent; ATE, aeration tank effluent; FCE, final clarification effluent; UV, UV treated effluent; DSA, downstream.

Table S8. Average colony counts (normalized to CFU/ml) for incubation temperatures and culturing media for.

a) combined total heterotrophic.

Temperature	LB	R2A	Temperature Avg
BT	5.00E+04	1.69E+05	1.09E+05
RT	2.45E+05	4.08E+05	3.27E+05
Media Avg	1.47E+05	2.89E+05	

b) and antibiotic-resistant counts.

Temperature	LB	R2A	Temperature Avg
BT	1.08E+04	2.31E+04	1.70E+04
RT	1.01E+05	1.41E+05	1.21E+05
Media Avg	5.58E+04	8.21E+04	

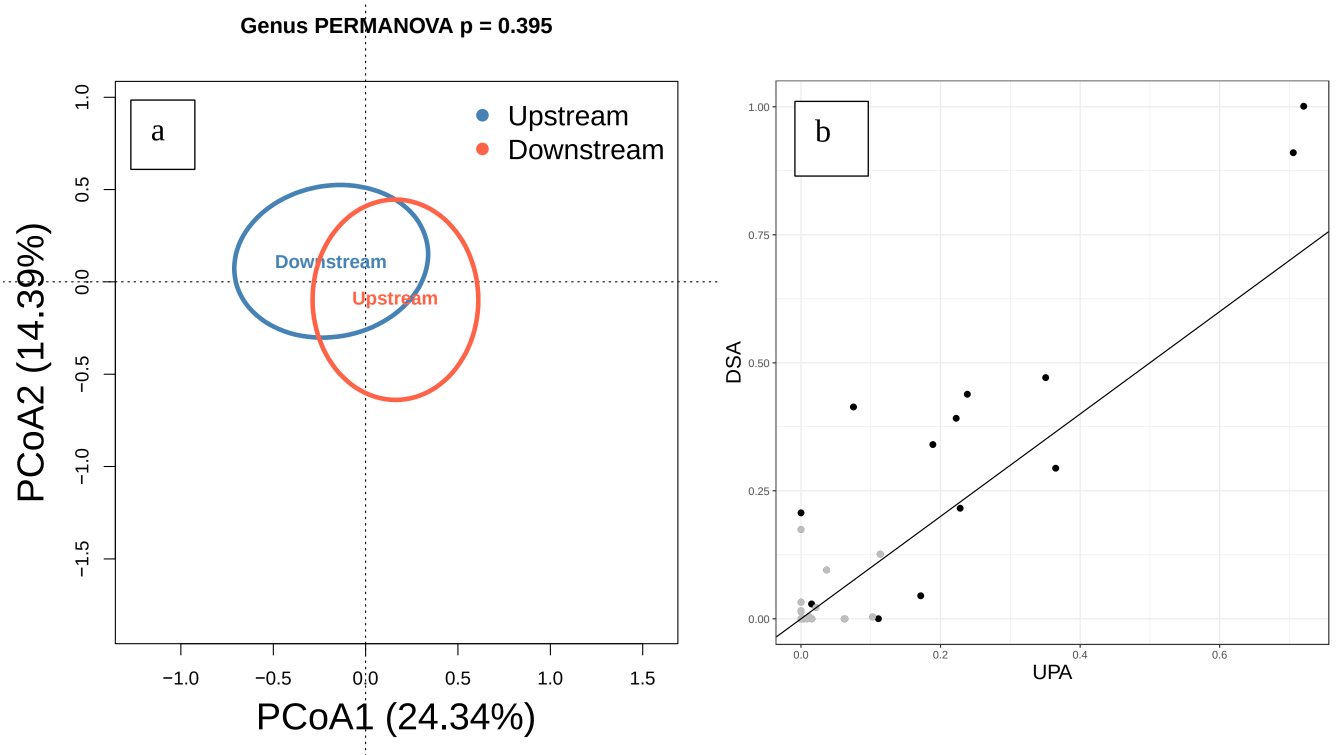

Figure S3 | a) No significant differences in beta diversities of the stream communities at the genus level from 16S rRNA gene sequencing (*p*=0.395). PCoA ordination for upstream (UPA) and downstream (DSA) communities with PC1 and PC2 components. Data colored by the two sampling locations. b) The mean relative abundance of each family from upstream and downstream catchment waters. No non-rare taxa were found to significantly differ between upstream and downstream waters using linear regression models at a 5% FDR threshold. Black symbols represent non-rare taxa found in 3% of all samples. Grey symbols represent rare taxa. The black line is the identity line (Upstream=Downstream).

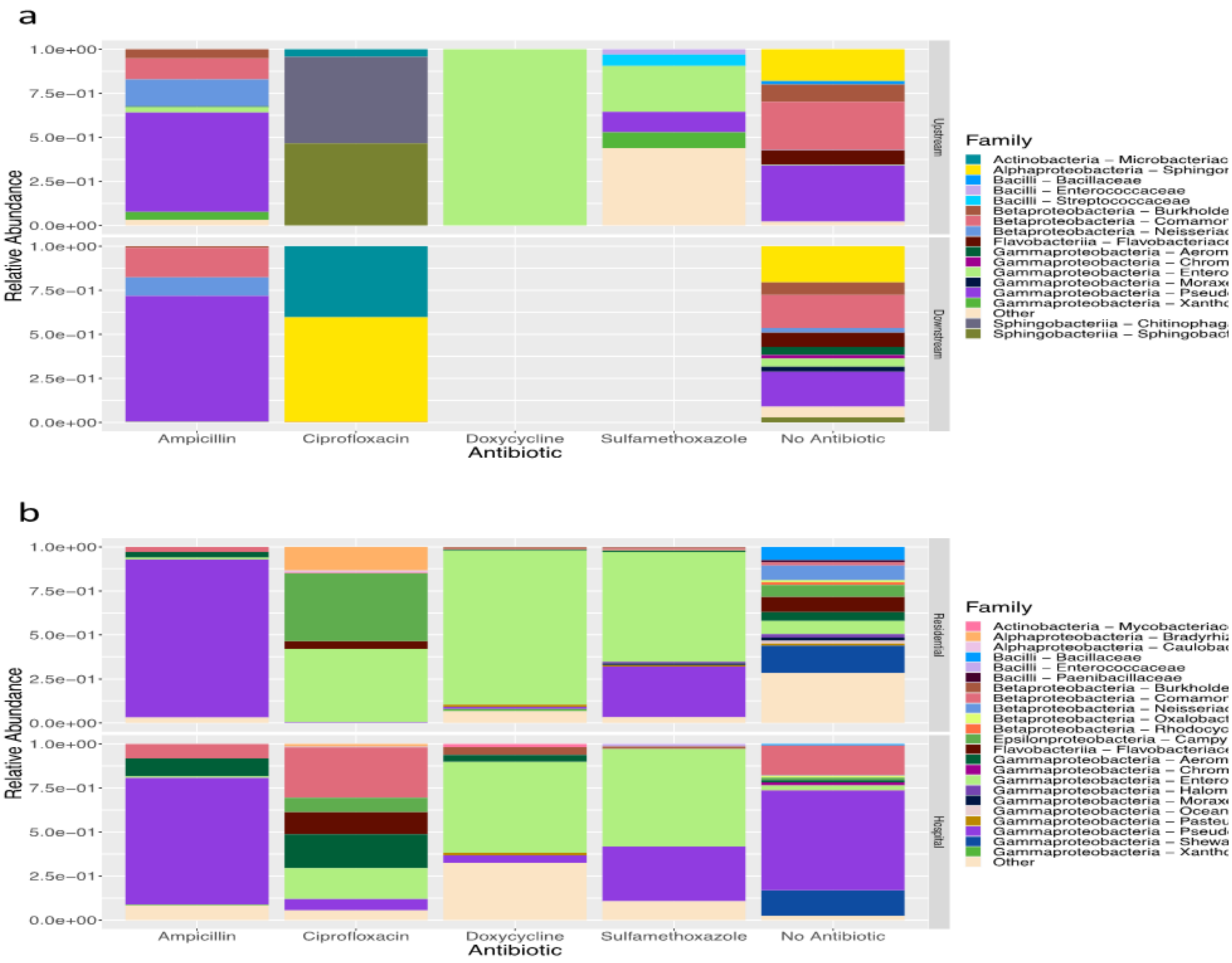

Figure S4. Relative abundance for total heterotrophic and antibiotic-resistant communities from a) upstream and downstream and b) residential and hospital sewage sampling locations. Combined Mallard and Sugar Creek samples. Families making up <1% of the total community at each site were excluded.

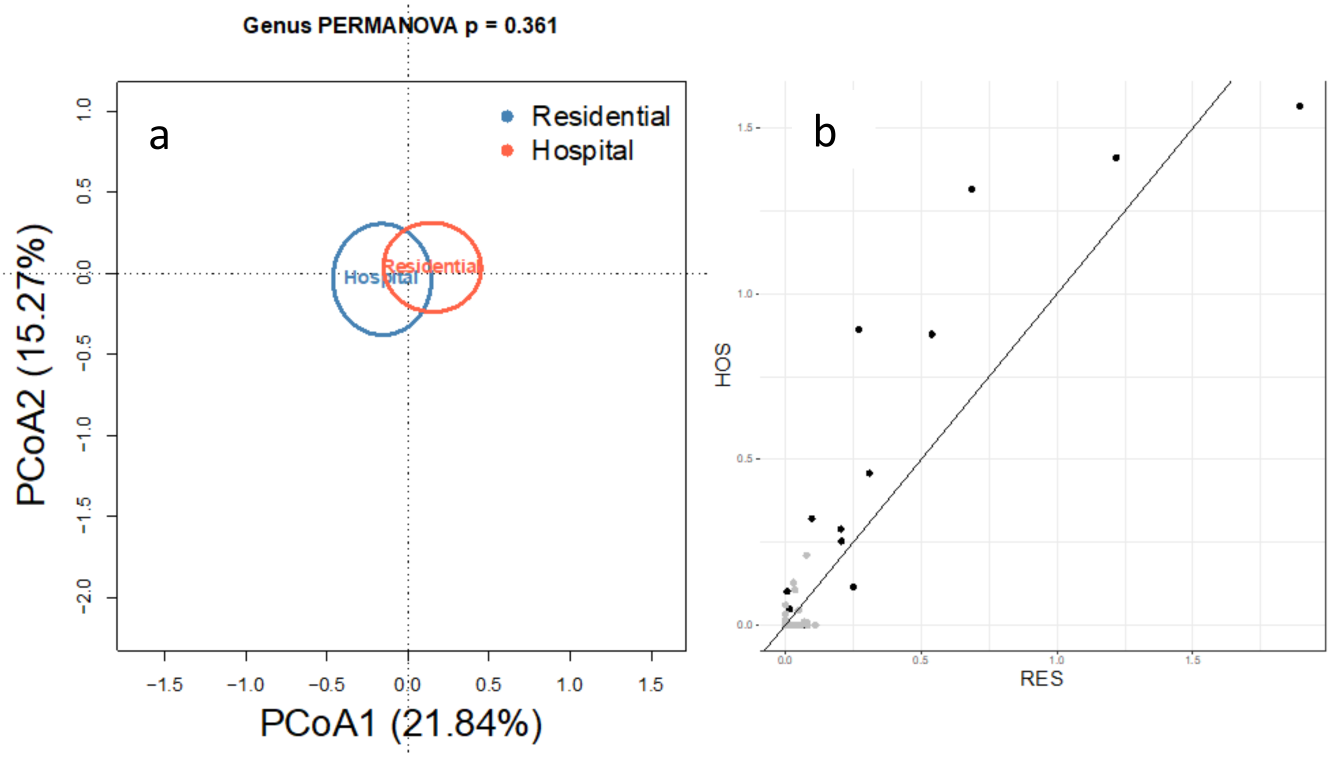

Figure S5. (**a**) No significant differences in beta diversities of the sewage communities at the genus level from 16S rRNA gene sequencing (*p*=0.361). PCoA ordination for residential (RES) and hospital (HOS) sewage communities with PC1 and PC2 components. Data colored by the two sampling locations. (**b**) The mean relative abundance of each family from residential and hospital sewage waters. No non-rare taxa were found to significantly differ between the sewage waters using linear regression models at a 5% FDR threshold. Block symbols represent non-rare taxa found in 3% of all samples. Grey symbols represent rare taxa. The black line is the identity line (Residential=Hospital).

Table S9. Average Shannon diversity value for incubation temperatures and culturing media for.

a) combined total heterotrophic.

Temperature	LB	R2A	Temperature Avg
BT	1.97	2.30	2.14
RT	1.79	2.27	2.00
Media Avg	1.88	2.28	

b) and antibiotic-resistant communities.

Temperature	LB	R2A	Temperature Avg
BT	1.80	2.06	2.14
RT	1.60	2.02	2.00
Media Avg	1.70	2.04	

Table S10. Differential abundance of taxa between culture media for total and combined antibiotic resistant communities.

a) Significant differential abundance *p* values for total heterotrophic and combined antibiotic resistant communities between two incubation temperatures classified to the genus level. Temperature column indicates the temperature at which the genus was found in greater abundance.

Phylum	Class	Family	Genus	Temperature	*p* value
Firmicutes	Bacilli	*Bacillaceae*	*Bacillus*	BT	0.024
Proteobacteria	Betaproteobacteria	*Burkholderiaceae*	*Burkholderia.Paraburkholderia*	BT	0.020
		Comamonadaceae	*Comamonas*	RT	0.013

Epsilonproteobacteria	*Campylobacteraceae*	*Arcobacter*	RT	0.001
Gammaproteobacteria	*Pseudomonadaceae*	*Pseudomonas*	RT	<0.001

b) Significant differential abundance *p* values for total heterotrophic and combined antibiotic resistant communities between two agar types classified to the genus level. Media column indicates the media in which the genus was found in greater abundance.

Phylum	Class	Family	Genus	Media	*p* value
Proteobacteria	Alphaproteobacteria	*Bradyrhizobiaceae*	*Bosea*	R2A	0.004
	Betaproteobacteria	*Burkholderiaceae*	*Polynucleobacter*	R2A	0.024
	Epsilonproteobacteria	*Campylobacteraceae*	*Arcobacter*	R2A	0.001
	Gammaproteobacteria	*Xanthomonadaceae*	*Stenotrophomonas*	R2A	0.025

c) Significant differential abundance *p* values for total heterotrophic and combined antibiotic resistant communities between two agar types classified to the family level. Media column indicates the media in which the family was found in greater abundance.

Phylum	Class	Family	Genus	Media	*p* value
Proteobacteria	Alphaproteobacteria	*Bradyrhizobiaceae*	*Bosea*	R2A	0.004
	Betaproteobacteria	*Burkholderiaceae*	*Polynucleobacter*	R2A	0.024
	Epsilonproteobacteria	*Campylobacteraceae*	*Arcobacter*	R2A	0.001
	Gammaproteobacteria	*Xanthomonadaceae*	*Stenotrophomonas*	R2A	0.025

Table S11. Differential abundance of taxa between cultured and environmental source samples. Sample Type indicates the DNA sample type in which the family was observed in greater abundance.

Phylum	Class	Family	Culture	Environmental	Sample Type	*p* value
Actinobacteria	Actinobacteria	*Nocardiaceae*	<0.01%	2.50%		n.s.
		Sporichthyaceae	<0.01%	7.90%		n.s.
		Microbacteriaceae	2.31%	2.64%		n.s.
Bacteroidetes	Cytophagia	*Cytophagaceae*	0.08%	5.99%		n.s.
	Flavobacteriia	*Cryomorphaceae*	<0.01%	3.27%		n.s.
		Flavobacteriaceae	2.37%	4.21%		n.s.
	Sphingobacteriia	*Chitinophagaceae*	0.66%	2.73%		n.s.
Firmicutes	Bacilli	*Bacillaceae*	1.66%	0.07%		n.s.
		Streptococcaceae	0.11%	2.15%		n.s.
	Clostridia	*Lachnospiraceae*	<0.01%	1.45%		n.s.
		Peptostreptococcaceae	<0.01%	1.23%	Environmental	<0.001
Proteobacteria	Alphaproteobacteria	*Caulobacteraceae*	1.65%	0.04%		n.s.
		Bradyrhizobiaceae	1.41%	0.02%		n.s.
	Betaproteobacteria	*Burkholderiaceae*	2.48%	6.13%		n.s.
		Comamonadaceae	8.97%	18.97%	Environmental	<0.001
		Neisseriaceae	3.25%	6.63%		n.s.
		Rhodocyclaceae	0.15%	4.13%	Environmental	<0.001
	Epsilonproteobacteria	*Campylobacteraceae*	1.47%	0.25%		n.s.
	Gammaproteobacteria	*Aeromonadaceae*	3.45%	1.59%		n.s.
		Enterobacteriaceae	32.16%	2.71%	Culture	<0.001
		Moraxellaceae	1.83%	6.42%	Environmental	0.001
		Pseudomonadaceae	17.93%	0.89%	Culture	<0.001
		Thiotrichaceae	<0.01%	7.54%		n.s.
		Xanthomonadaceae	1.10%	0.14%		n.s.
Other	Other	Other	16.96%	10.40%		n.s.

n.s. – not significant.

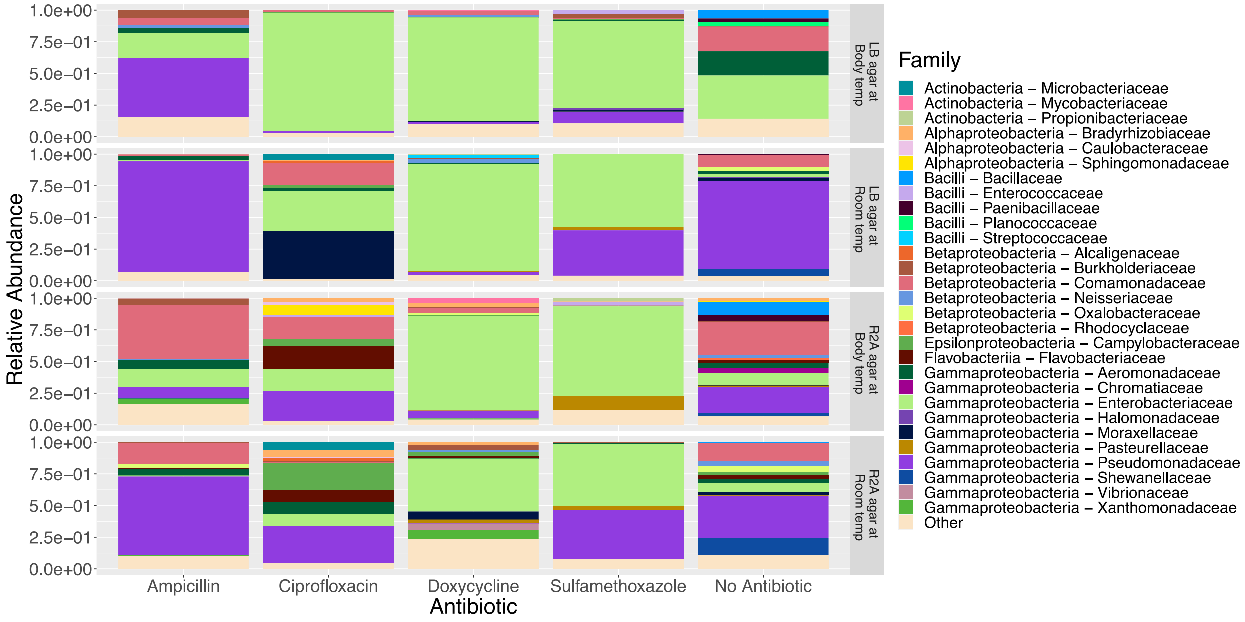

Figure S6. | Relative abundance of taxonomic families grown under four incubation conditions with antibiotic-amended media. Families making up <1% of the total community at each site were excluded.
